# Assessment of groundwater quality by water quality indices for irrigation and drinking in South West Delhi, India

**DOI:** 10.1016/j.dib.2018.04.120

**Published:** 2018-05-03

**Authors:** Sanigdha Acharya, S.K. Sharma, Vinita Khandegar

**Affiliations:** University School of Chemical Technology, India; Guru Gobind Singh Indraprastha University, Dwarka, New Delhi, India

**Keywords:** Groundwater, Water quality indices, Irrigation, Drinking, Delhi

## Abstract

Groundwater quality should be continuously monitored for irrigation and drinking purpose so that risk from geochemical contaminants can be reduced by appropriate treatment method. Therefore, the focus of the present study was to determine the suitability of groundwater collected from South West Delhi, India, for irrigation and drinking purpose on the basis of various water quality indices. In order to assess the groundwater quality, 50 samples were collected from different sites of selected study area and parameters such as pH, EC (electrical conductivity), total dissolved solids (TDS), salinity, total hardness (TH), total alkalinity (HCO_3_^−^), calcium (Ca^+2^), magnesium (Mg^+2^), sodium (Na^+^), potassium (K^+^), chloride (Cl^−^), Fluoride (F^−^), sulfates (SO_4_^−2^) and Nitrates (NO_3_^−^) were determined. Based on the above parameters, sodium adsorption ratio (SAR), soluble sodium percentage (SSP), residual sodium carbonate (RSC), permeability index (PI), magnesium adsorption ratio (MAR), Kelley's ratio (KR) and Na% were calculated. Water quality index (WQI), which is an important and unique rating to represent the overall water quality in a single term that is useful to determine the suitability of water for human consumption, was also estimated. The present dataset demonstrated the application of water quality indices that would be helpful to policymakers for appropriate management, treatment and sustainable societal development at large.

**Specifications Table**

**Table:** 

Subject area	cGroundwater study
More specific subject area	Environmental Science
Type of data	Table and Figure
How data was acquired	Water analysis kit (NPC363D, India), UV–vis Double Beam spectrophotometer (Hitachi U-2900, India), Flame photometer (Toshniwal TMF-45, India).
Data format	Raw, analyzed
Experimental factor	Groundwater samples from 50 different locations in South-West Delhi, India were collected.
Experimental features	Parameters such as EC, TH, HCO_3_^−^, Ca^+2^, Mg^+2^, Na^+^, K^+^, F^−^, Cl^−^, SO_4_^−2^ and NO_3_^−^ were analyzed according to APHA method.
Data source location	South-West Delhi, New Delhi, India
Data accessibility	This article contains Water Quality Indices dataset.

**Value of the data**

• This dataset gives an idea about the Water Quality Indices of the studied area which helps to the decision-makers in order to understand the status of the groundwater quality for irrigation and drinking purpose.
• Anions and cations are one of the most common parameters of water resources; hence their incessant monitoring is very important. The water quality indices such as SAR, MAR, SSP, RSC, PI, Na% and KR were calculated to evaluate the suitability of the groundwater studied for agricultural purposes.
• Piper diagram and WQI calculations were used to determine the suitability of drinking water for the studied area. The WQI values indicated that 34% of the samples were in the range of good water and 66% of the samples were in the range of poor to unsuitable for drinking category.
• This dataset can be used as a tool to identify the process and mechanisms affecting the chemistry of groundwater in the study area.

## 1 Data

**Fig. 1:**
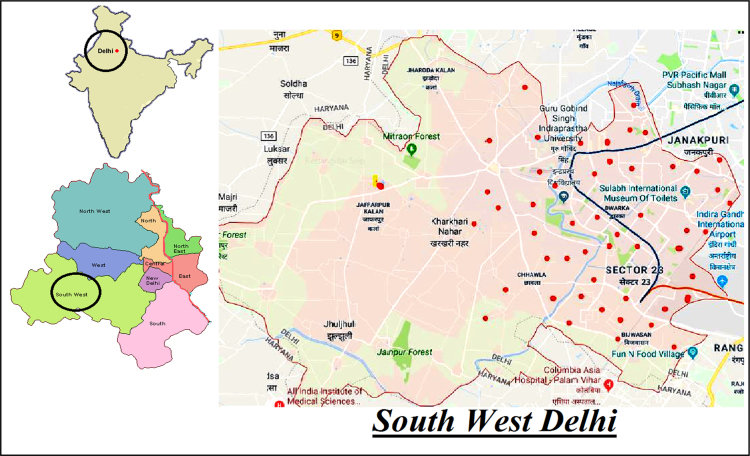
Sampling points of the *South West Delhi*, India.

**Fig. 2:**
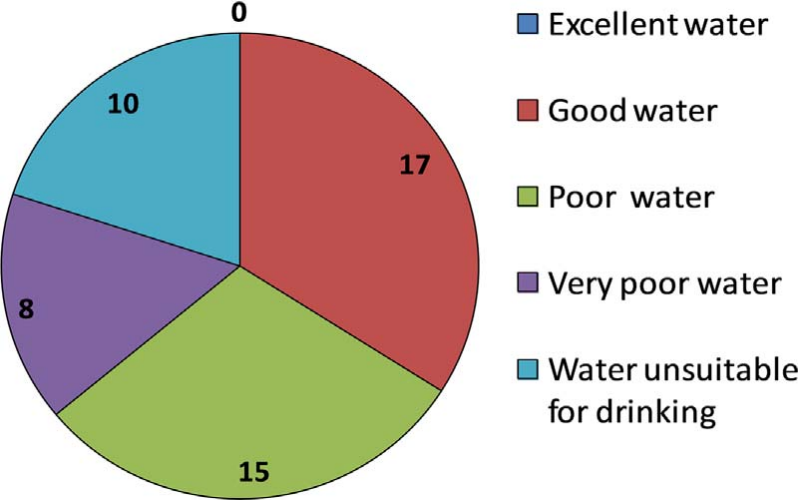
Results of WQI for drinking purpose.

**Fig. 3:**
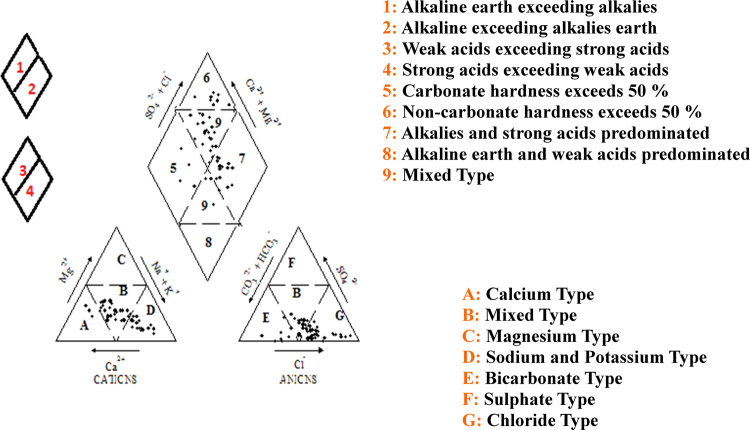
Piper trilinear diagram for groundwater samples of the study area.

**Fig. 4:**
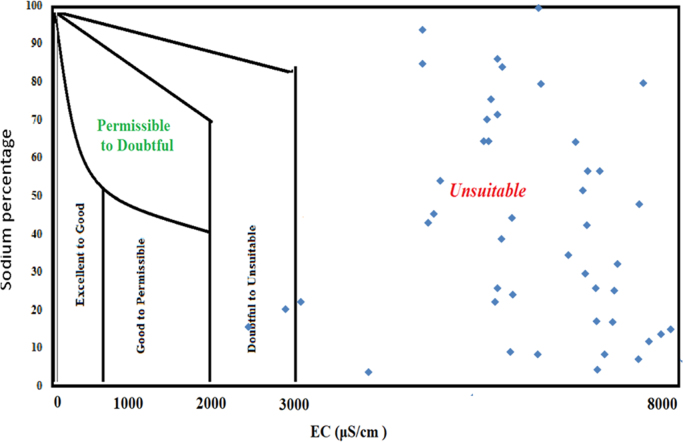
Wilcox diagram based on Sodium percent Vs EC.

This dataset contains 7 Tables and 4 Figs. that represent the qualityof the groundwater for irrigation and drinking purposes of South West Delhi,India. Fig. 1 shows the sampling points of the studied area. Table 1 depictsthe milliequivalent (meq/L) values of parameters used to determine waterquality indices. The criteria and summary of water quality indices forirrigation purpose are tabulated in Table 2 and Table 3 respectively. Grades of groundwater samples for irrigation purpose based on variousindices with their ranges are given in Table 4. The parameters for calculation of WQI with BISstandards [5] are shown in Table 5. The range of WQI for drinking water inIndia and results of analyzed samples in studied area are given in Table 6 and Fig. 2. Table 7 shows the Pearson correlation among various parameters. Piper trilinear diagram is represented in Fig. 3. Wilcox diagram has been plotted between thesodium percentage and EC (Fig. 4).

**Table 1:** Values of anion and cations in meq/L for the present study.

**Sample number**	**Na**^**+1**^**(meq/L)**	**k**^**+1**^**(meq/L)**	**Ca**^**+2**^**(meq/L)**	**Mg**^**+2**^**(meq/L)**	**HCO**_**3**_^**−1**^**(meq/L)**	**Cl**^**−1**^**(meq/L)**	**No**_**3**_^**−1**^**meq/L**	**F**^**−1**^**(meq/L)**	**So**_**4**_^**−2**^**(meq/L)**	**pH**	**TDS (mg/L)**	**Salinity (mg/L)**
S1	22.35	0.21	10.09	16.47	4.34	9.06	0.15	0.06	2.08	7.18	2535	4910
S2	28.26	0.72	47.20	34.43	5.41	14.13	0.23	0.05	2.50	6.51	5645	10,190
S3	14.52	0.31	6.23	3.77	3.84	12.86	0.21	0.04	1.48	6.92	1725	2415
S4	11.13	0.08	3.05	3.37	3.28	11.86	0.19	0.02	1.23	7.24	1300	1820
S5	8.70	0.49	3.88	5.06	1.80	3.16	0.05	0.03	0.98	7.35	1230	1722
S6	6.04	0.18	1.19	1.70	1.64	2.84	0.05	0.04	0.79	7.16	800	1120
S7	6.70	0.28	4.09	4.65	1.98	4.36	0.07	0.02	0.92	7.35	900	1260
S8	8.13	0.26	4.90	3.28	2.34	2.16	0.03	0.04	1.17	6.96	1194.5	1672.3
S9	8.70	0.28	1.95	1.61	2.36	4.52	0.07	0.03	1.48	7.45	1703	2384.2
S10	10.65	0.15	20.53	19.99	4.33	70.62	1.14	0.04	2.29	7.05	4015	5621
S11	7.74	0.15	1.72	2.95	5.66	4.96	0.08	0.05	0.87	7.52	800	1120
S12	9.26	0.28	4.46	4.87	7.87	17.98	0.29	0.03	2.04	7.39	2160	3024
S13	10.00	0.33	16.03	24.85	5.66	40.55	0.65	0.10	2.75	7.96	3770	5278
S14	9.26	0.15	20.84	27.68	7.54	86.24	1.39	0.08	2.66	7.80	3250	4550
S15	1.17	0.18	2.15	1.83	2.54	2.47	0.04	0.06	0.00	6.89	363	508.2
S16	1.04	0.21	10.22	8.80	5.16	20.52	0.33	0.02	0.00	7.49	170	238
S17	5.22	0.28	3.88	6.25	5.16	10.59	0.17	0.03	1.81	6.98	1340	1876
S18	33.91	0.90	29.91	42.88	3.61	131.77	2.13	0.10	4.99	7.16	7890	11,046
S19	7.17	0.31	2.20	2.31	6.64	8.64	0.14	0.05	1.85	7.60	1345	1883
S20	2.13	0.23	2.05	1.82	7.54	5.74	0.09	0.02	0.00	7.89	829	1160.6
S21	1.00	0.18	4.41	2.68	5.90	5.33	0.09	0.03	0.15	7.58	565	791
S22	8.57	0.69	20.14	27.69	6.07	50.28	0.81	0.04	2.50	7.55	4360	6104
S23	7.13	0.31	18.09	18.09	6.31	46.55	0.75	0.04	2.25	7.87	2610	3654
S24	5.35	0.36	9.12	10.87	4.10	47.68	0.77	0.05	2.33	7.14	2750	3850
S25	1.00	0.33	2.25	1.94	6.97	2.54	0.04	0.02	0.48	7.69	329.5	461.3
S26	2.35	0.28	1.70	2.48	5.98	10.90	0.18	0.05	0.81	7.17	1433.5	2006.9
S27	1.96	0.18	10.62	7.94	7.54	17.95	0.29	0.04	0.46	6.78	1115	1561
S28	6.65	0.33	9.94	14.52	7.30	33.26	0.54	0.03	0.98	7.59	2485	3479
S29	1.00	0.03	1.95	1.82	5.90	10.03	0.16	0.03	0.67	7.69	259	362.6
S30	8.70	0.28	8.27	6.72	7.30	19.13	0.31	0.04	2.21	6.90	2329.5	3261.3
S31	3.00	0.21	5.45	3.44	5.66	15.69	0.25	0.02	0.48	7.70	1435	2009
S32	2.52	0.18	4.45	3.53	5.57	15.21	0.25	0.04	0.94	8.00	1280	1792
S33	8.00	0.41	22.28	27.00	7.26	23.55	0.38	0.03	1.62	7.45	2493.5	3490.9
S34	3.87	0.23	10.44	8.99	7.11	24.81	0.40	0.04	1.31	8.20	1415.5	1981.7
S35	11.30	0.54	4.46	3.50	5.66	15.37	0.25	0.02	2.79	7.40	3360	4704
S36	7.78	0.36	9.39	7.38	7.11	22.81	0.37	0.00	5.35	7.52	2065	2891
S37	2.04	0.23	3.03	2.98	6.16	8.84	0.14	0.00	3.47	7.67	1300	1820
S38	6.78	0.26	2.88	2.37	7.14	8.73	0.14	0.01	4.87	7.38	1250	1750
S39	15.48	0.59	10.48	12.95	14.44	25.64	0.41	0.03	7.18	7.65	3270	4578
S40	7.04	0.31	3.42	2.65	6.85	11.83	0.19	0.02	1.10	7.54	1305	1827
S41	7.61	0.36	4.45	4.92	6.65	13.77	0.22	0.02	4.16	7.98	2340	3276
S42	5.22	0.26	2.73	1.93	4.53	10.34	0.17	0.03	1.62	6.86	1215	1701
S43	3.43	0.28	3.15	2.62	3.58	10.34	0.17	0.05	1.35	7.68	859	1202.6
S44	10.00	0.44	12.63	15.36	8.30	20.27	0.33	0.03	5.58	7.98	2777	3887.8
S45	7.61	0.33	8.83	9.93	6.86	20.48	0.33	0.04	0.67	7.90	2310	3234
S46	6.87	0.36	3.94	5.66	11.06	13.18	0.21	0.04	0.96	8.25	2270	3178
S47	4.35	0.26	10.46	11.96	8.76	22.59	0.36	0.04	1.12	7.22	1691.5	2368.1
S48	8.61	0.67	15.52	23.70	6.73	20.27	0.33	0.05	1.39	8.06	3489	4884.6
S49	9.13	0.46	8.90	12.09	6.73	18.03	0.29	0.02	2.46	8.50	2835	3969
S50	6.70	0.28	8.43	9.92	5.30	18.75	0.30	0.03	2.81	7.60	2462.5	3447.5

**Table 2:** Summary of water quality indices for irrigation [2], [3].

**Indices**	**Acronym**	**Formula**
Sodium absorption ratio	SAR	$\mathrm{SAR}=\frac{\mathrm{Na}}{\sqrt{(\mathrm{Ca}+\mathrm{Ma})/2}}$
Residual sodium carbonate	RSC	${(\mathrm{Co}}_{3}+{\mathrm{HCo}}_{3})+(\mathrm{Ca}+\mathrm{Mg})$
Soluble sodium percentage	SSP	$\left(\frac{\mathrm{Na}}{\mathrm{Ca}+\mathrm{Mg}+\mathrm{Na}}\right)*100$
Kelly Ratio	KR	$\frac{\mathrm{Na}}{\mathrm{Ca}+\mathrm{Mg}}$
Sodium percentage	Na%	$\left(\frac{\mathrm{Na}+K}{\mathrm{Ca}+\mathrm{Mg}+\mathrm{Na}+K}\right)*100$
Magnesium hazard	MH	$\left(\frac{\mathrm{Mg}}{\mathrm{Ca}+\mathrm{Mg}}\right)*100$
Permeability index	PI	$\left(\frac{\mathrm{Na}+K+\sqrt{\mathrm{HC}o_{3}}}{\mathrm{Ca}+\mathrm{Mg}+\mathrm{Na}+K}\right)*100$

**Table 3:** Results of water quality indices for irrigation.

**Sample number**	**SAR**	**RSC**	**SSP**	**KR**	**Na%**	**MH**	**PI**
S1	6.13	−22.22	46.11	0.84	45.92	62.01	50.16
S2	4.42	−76.22	26.37	0.35	26.20	42.18	28.30
S3	6.49	−6.17	60.47	1.45	59.72	37.68	67.61
S4	6.22	−3.13	63.89	1.74	63.61	52.51	73.89
S5	4.11	−7.13	52.08	0.97	50.68	56.64	58.09
S6	5.02	−1.25	69.63	2.09	68.26	58.88	82.30
S7	3.20	−6.75	45.22	0.77	44.41	53.24	53.37
S8	4.02	−5.84	51.41	0.99	50.61	40.13	59.85
S9	6.52	−1.20	73.25	2.44	71.60	45.12	83.86
S10	2.37	−36.19	21.12	0.26	21.05	49.33	25.11
S11	5.06	0.98	63.60	1.66	62.82	63.09	81.75
S12	4.29	−1.46	51.32	0.99	50.55	52.22	65.42
S13	2.21	−35.23	20.31	0.24	20.18	60.79	24.82
S14	1.88	−40.98	16.29	0.19	16.25	57.05	20.99
S15	0.83	−1.44	26.27	0.30	25.38	45.88	55.28
S16	0.34	−13.85	6.22	0.05	6.16	46.27	17.37
S17	2.32	−4.96	35.84	0.52	35.19	61.72	49.74
S18	5.62	−69.18	32.62	0.47	32.35	58.91	34.12
S19	4.77	2.12	64.00	1.59	62.36	51.23	83.84
S20	1.53	3.66	39.31	0.55	37.85	47.04	81.87
S21	0.53	−1.19	14.58	0.14	14.26	37.75	43.64
S22	1.75	−41.76	16.42	0.18	16.22	57.89	20.53
S23	1.68	−29.87	17.17	0.20	17.05	50.01	22.81
S24	1.69	−15.90	22.52	0.27	22.20	54.38	30.08
S25	0.69	2.78	25.70	0.24	24.15	46.21	71.94
S26	1.62	1.80	40.28	0.56	38.61	59.28	74.53
S27	0.64	−11.02	10.41	0.11	10.32	42.79	23.59
S28	1.90	−17.16	22.45	0.27	22.22	59.37	30.81
S29	0.73	2.13	21.49	0.27	21.38	48.22	72.01
S30	3.18	−7.70	37.89	0.58	37.45	44.84	48.71
S31	1.42	−3.23	26.97	0.34	26.51	38.67	46.18
S32	1.26	−2.41	25.72	0.32	25.29	44.24	47.39
S33	1.61	−42.02	14.68	0.16	14.58	54.78	19.25
S34	1.24	−12.32	17.60	0.20	17.43	46.28	28.76
S35	5.67	−2.30	61.48	1.42	59.81	43.96	71.82
S36	2.69	−9.66	33.16	0.46	32.68	43.99	43.39
S37	1.18	0.14	28.22	0.34	27.44	49.62	57.38
S38	4.19	1.90	58.54	1.29	57.32	45.16	79.07
S39	4.52	−8.99	41.30	0.66	40.68	55.29	50.30
S40	4.05	0.78	56.09	1.16	54.80	43.64	74.31
S41	3.52	−2.72	46.94	0.81	45.96	52.51	60.84
S42	3.42	−0.12	55.45	1.12	54.04	41.46	75.06
S43	2.02	−2.18	40.42	0.60	39.22	45.41	59.18
S44	2.67	−19.68	27.47	0.36	27.16	54.88	34.66
S45	2.48	−11.91	30.11	0.41	29.74	52.92	39.54
S46	3.14	1.46	43.89	0.72	42.96	58.96	62.72
S47	1.30	−13.66	17.20	0.19	17.04	53.36	27.99
S48	1.94	−32.49	19.39	0.22	19.13	60.43	24.48
S49	2.82	−14.26	31.84	0.43	31.36	57.58	39.85
S50	2.21	−13.05	27.86	0.36	27.55	54.07	36.64

**Table 4:** Grades of groundwater samples for irrigation purpose based on various indices.

**Parameters**	**Range**	**Water class**	**No. of samples**	**Samples (%)**
EC	< 250	Excellent	0	0.00
	250–750	Good	4	8.00
	750–2250	Permissible	7	14.00
	> 2250	Doubtful	39	78.00
SAR	0–10	Excellent	50	100
	10–18	Good	0	0
	18–26	Doubtful	0	0
	>26	Unsuitable	0	0
RSC	< 1.25	Good	43	86
	1.25–2.5	Doubtful	5	10
	>2.5	Unsuitable	2	4
KR	< 1	Suitable	40	80
	> 2	Unsuitable	10	20
SSP	< 50	Good	37	74
	> 50	Unsuitable	13	26
PI	< 80	Good	5	10
	80–100	Moderate	45	90
	100–120	Poor	0	0
MH	< 50	Suitable	23	46
	50.00	Harmful and Unsuitable	27	54
Na%	< 20	Excellent	10	20
	20–40	Good	22	44
	40–60	Permissible	13	26
	60–80	Doubtful	5	10
	> 80	Unsuitable	0	0
T.H	< 75	Soft	0	0
	75–150	Moderately Hard	3	6
	150–300	Hard	6	12
	> 300	Very Hard	41	82

**Table 5:** Assigned and relative weight for WQI computation with BIS standards [4], [5].

**S.N.**	**Parameters**	**BIS standards desired limit**	**Weight (*w_i_*)**	**Relative weight (*RW****_**i**_***)**
1	pH	6.5–8.5	4	0.13
2	TDS	500	4	0.13
3	Hardness	300	3	0.10
4	Calcium	75	3	0.10
5	Magnesium	30	3	0.10
6	Nitrate	45	4	0.13
7	Chlorides	250	2	0.06
8	Sulphate	200	2	0.06
9	Fluoride	1	4	0.13
10	Total Alkalinity	200	2	0.06
		Total	31	1.00

*All units in mg/L except pH.

**Table 6:** Range and classification of WQI for drinking purpose in the present study.

**S.N.**	**WQI value**	**Water Quality**	**No. of water samples**	**% of samples**
1	< 50	Excellent water	0	0
2	50–100	Good water	17	34
3	100–200	Poor water	15	30
4	200–300	Very poor water	8	16
5	> 300	Unsuitable for drinking	10	20

**Table 7:** Pearson correlation coefficient among various parameters.

**Parameter**	**Temp (°C)**	**pH**	**EC (µS/cm)**	**TDS (mg/L)**	**Salinity (mg/L)**	**Hardness (mg/L)**	**Sodium mg/L)**	**Potassium (mg/L)**	**Calcium (mg/L)**	**Magnesium (mg/L)**	**Nitrate (mg/L)**	**Fluoride (mg/L)**	**Sulphate mg/L)**	**Chlorides (mg/L)**	**Alkalinity (mg/L)**
**Temp (°C)**	1.00														
**pH**	0.10	1.00													
**EC (µS/cm)**	0.16	−0.04	1.00												
**TDS (mg/L)**	0.16	−0.04	1.00	1.00											
**Salinity (mg/L)**	0.18	−0.10	0.99	0.99	1.00										
**Hardness (mg/L)**	0.15	−0.03	0.89	0.89	0.91	1.00									
**Sodium (mg/L)**	0.01	−0.27	0.81	0.81	0.85	0.71	1.00								
**Potassium (mg/L)**	0.03	0.03	0.77	0.77	0.76	0.66	0.64	1.00							
**Calcium (mg/L)**	0.16	−0.16	0.81	0.81	0.85	0.96	0.66	0.59	1.00						
**Magnesium (mg/L)**	0.14	−0.01	0.87	0.87	0.87	0.94	0.67	0.64	0.92	1.00					
**Nitrate (mg/L)**	−0.01	0.15	−0.05	−0.05	−0.08	−0.05	−0.11	0.11	−0.15	−0.13	1.00				
**Fluoride (mg/L)**	0.13	−0.12	0.51	0.51	0.51	0.48	0.46	0.23	0.45	0.59	−0.05	1.00			
**Sulphate (mg/L)**	−0.20	0.09	0.55	0.55	0.52	0.44	0.49	0.48	0.31	0.37	0.20	−0.01	1.00		
**Chlorides (mg/L)**	0.09	0.02	0.76	0.76	0.69	0.68	0.49	0.40	0.62	0.77	−0.16	0.57	0.38	1.00	
**Alkalinity (mg/L)**	−0.03	0.42	0.13	0.13	0.11	0.17	−0.05	0.16	0.10	0.12	0.29	−0.16	0.38	0.07	1.00

## 2 Experimental design, materials, and methods

### 2.1 Study area description

The South West District, Delhi stretches over an area of 420 square kilometers approximately. It is one of the eleven administrative districts of the National Capital Territory of Delhi in India. The Subcity of Dwarka serves as the administrative headquarters of *South West Delhi*. The sampling sites were chosen to cover the entire studied area (Fig. 1).

### 2.2 Analytical procedures

All sampling steps and data analysis were performed according to standard methods for water and wastewater [1]. EC, pH, and TDS were recorded using water analysis kit (NPC363D, India). The concentrations of nitrates and sulphate were determined using UV–vis Spectrophotometer (Hitachi U-2900, India). Calcium and magnesium were measured by EDTA titrimetric method. Chloride by standard AgNO_3_ titration and bicarbonate by titration with HCl. Sodium, potassium by flame photometer (Toshniwal TMF-45, India) and fluoride was determined using SPANDS method.

### 2.3 Data treatment and classification methods

#### 2.3.1 Water quality indices calculation for irrigation

The overall irrigational water quality of the collected samples was assessed using water quality indices such as SAR, MAR SAR, MAR, SSP, RSC, PI, Na % and KR using Table 1 and Table 2.

#### 2.3.2 Water quality index calculation for drinking

WQI is a valuable and unique parameter for identifying the water quality and its sustainability for drinking purposes. It represents the composite influence of different water quality parameters and provides water quality information to legislative decision makers and the general masses.

The groundwater quality index(WQI) for drinking purpose is calculated by the following steps:

1. Weight is assigned to the parameters under consideration (*w_i_*). These weights indicate the relative harmfulness when present in water. The maximum weight assigned is five and minimum is one. The relative weights (*RW_i_*) are calculated as per the formula
  (1) $$RW_{i}=\frac{w_{i}}{\sum_{1}^{n}w_{i}}$$
  where *n* is the number of parameters being assessed by WQI.
2. Each parameter is assigned a quality rating scale (*q_i_*) as per the formula
  (2) $$q_{i}=\frac{e_{i}\;-\;v_{i}}{b_{i}\;-\;v_{i}}\times 100$$
  where *e_i_* is the value of each parameter as observed experimentally, v_i_ is the base value for each parameter (0 for all parameters except pH (7)), *b_i_* is the standard value as recommended by BIS [5].
3. The sub-index (*S.I._i_*) of each parameter for a place is thus calculated as
  (3) $$S.{I.}_{i}=q_{i}\times {\mathrm{RW}}_{i}$$
4. WQI of each station is calculated as
  (4) $$WQI=\sum_{1}^{n}S.I._{i}$$

#### 2.3.3 Piper and Wilcox diagram

The hydrochemical evolution of groundwater can be understood by plotting Piper Trilinear diagram for the major cations and anions present in groundwater (Fig. 3). Wilcox diagram is used to determine classification and viability of groundwater for irrigation purposes based on sodium percent and EC (Fig. 4).
