# Supplementary material for: Assessment of groundwater quality by water quality indices for irrigation and drinking in South West Delhi, India
Source: Data Brief. 2018 May 3;18:2019–28. doi: 10.1016/j.dib.2018.04.120 (PMC5998705; doi:10.1016/j.dib.2018.04.120)
Supplement: Supplementary file 1 — Supplementary material [file mmc1.docx]

**Conflict of interest**

All the author confirms no Conflict of Interest.
